# Age-related decline in the expression of *GDF9* and *BMP15* genes in follicle fluid and granulosa cells derived from poor ovarian responders

**DOI:** 10.1186/s13048-020-00757-x

**Published:** 2021-01-04

**Authors:** Yan Gong, Jesse Li-Ling, Dongsheng Xiong, Jiajing Wei, Taiqing Zhong, Hao Tan

**Affiliations:** 1 Reproductive Medicine Center, Sichuan Provincial Women’s and Children’s Hospital, The Affiliated Women’s and Children’s Hospital of Chengdu Medical College, 290 Shayan West Second Street, Wuhou District, Chengdu, 610045 Sichuan China; 2 State Key Laboratory of Biotherapy, West China Hospital, Sichuan University, 37 Guoxuexiang, Wuhou District, Chengdu, 610041 Sichuan China; 3 Laboratory Medicine Center, Sichuan Provincial Women’s and Children’s Hospital, The Affiliated Women’s and Children’s Hospital of Chengdu Medical College, Chengdu, 610045 Sichuan China; 4 Department of Genetics, School of Bioscience and Technology, Chengdu Medical College, Chengdu, 610500 Sichuan China

**Keywords:** *GDF9* gene, *BMP15* gene, Poor ovarian response, In vitro fertilization, Age

## Abstract

**Background:**

Growth differentiation factor 9 (*GDF9*) and bone morphogenetic protein 15 (*BMP15*) genes play important roles in folliculogenesis. Altered expression of the two have been found among patients with poor ovarian response (POR). In this prospective cohort study, we have determined the expression of the *GDF9* and *BMP15* genes in follicle fluid (FF) and granulosa cells (GCs) derived from poor ovarian responders grouped by age, and explored its correlation with the outcome of in vitro fertilization and embryo transfer (IVF-ET) treatment.

**Methods:**

A total of 196 patients with POR were enrolled from a tertiary teaching hospital. The patients were diagnosed by the Bologna criteria and sub-divided into group A (< 35 year old), group B (35–40 year old), and group C (> 40 year old). A GnRH antagonist protocol was conducted for all patients, and FF and GCs were collected after oocyte retrieval. Expression of the *GDF9* and *BMP15* genes in the FF and GCs was determined with enzyme-linked immunosorbent assay (ELISA), quantitative real-time polymerase chain reaction (qRT-PCR) and Western blotting.

**Results:**

Compared with group C, groups A and B had significantly more two pronuclei (2PN) oocytes and transplantable embryos, in addition with higher rates of implantation and clinical pregnancy (*P* <  0.05). The expression level of *GDF9* and *BMP15* genes in the FF and GCs differed significantly among the three groups (*P* <  0.05), showing a trend of decline along with age. The ratio of GDF9/BMP15 mRNA levels were similar among the three groups (*P* > 0.05). The relative levels of GDF9 and BMP15 proteins in GCs have correlated with the relative mRNA levels in GCs and protein concentrations in FF (*P* <  0.05).

**Conclusions:**

For poor ovarian responders, in particular those over 40, the expression of *GDF9* and *BMP15* is declined along with increased age and in accompany with poorer oocyte quality and IVF outcome, whilst the ratio of GDF9/BMP15 mRNA levels remained relatively constant.

**Trial registration:**

Chinese Clinical Trial Registry Center (ChiCTR1800016107). Registered on 11 May 2018.

## Introduction

Poor ovarian response (POR) poses a great challenge for in vitro fertilization and embryo transfer (IVF-ET) treatment. Patients with POR have fewer retrieved oocytes, fewer transferable embryos, lower pregnancy rates, and greater odds for cycle cancellation and miscarriage [1]. With a prevalence of 9% ~ 24% in various IVF centers [2], POR affects approximately 11.9% of Chinese women undergoing IVF-ET treatment [3]. Although various stimulation protocols and strategies have been proposed, patients with POR still benefited little from such treatment [4].

According to the Bologna criteria, POR is diagnosed with at least two of the following three criteria: advanced maternal age or any other risk factor for POR; previous history of POR; and an abnormal ovarian reserve test [5]. Although above criteria are useful for predicting ovarian response and counseling purpose, it has grouped together women with various etiologies and clinical characteristics [6]. Some have reported that, compared with elder patients, younger poor ovarian responders (< 40, especially < 35 year old) have higher embryo quality and pregnancy rate but lower miscarriage rate [7–9]. Age has therefore been proposed as an independent predictor for POR [7, 8]. Studies have shown that elder poor responders, in particular those over 40, have significantly lower cumulative live birth rate (CLBR) compared with young poor responders (< 35) [10–12]. Therefore, 35 and 40 years old have been used as the thresholds by the Poseidon Criteria and Bologna Criteria, respectively [5, 6]. For elder poor responders, age-related infertility and decreased ovarian reserve (DOR) are the main obstacles, whereas for younger poor responders, ovarian aging seems to be independent of age. The heterogeneous prognosis may be attributable to various etiology of the patients.

Risk factors for POR include fecundity decline with age, ovarian cystectomy, chronic smoking, genetic factors, previous chemotherapy and/or radiotherapy, etc. [13]. The pathophysiology of POR is complex and may involve declined endocrine feedback of ovarian factors [14–16]. Oocyte-secreted factors (OSFs) can regulate follicle development [17]. The transforming growth factor β (TGFβ) superfamily is one of the most important OSFs which control the function of granulosa cells (GCs). The latter in turn can nourish the oocyte [18]. Growth differentiation factor 9 (*GDF9*) and bone morphogenetic protein 15 (*BMP15*, also known as *GDF9B*) are essential members of the TGF-β superfamily which can enhance the proliferation and metabolism of the GCs and stimulate the expression of kit ligand (KL) in GCs. The GCs can then provide nutrients, steroid hormones and growth factors which can enable oocyte maturation via an intimate cross-talk [15, 17]. Cumulus expansion is a complex process which is critical for oocyte maturation and ovulation [19]. The process is highly dependent on the interactions of two signals released by epidermal growth factor (EGF)-like peptides and OSFs [20]. Previous studies have shown that genetic variants and abnormal expression of *GDF9* and *BMP15* may predispose to follicle atresia and early exhaustion of ovarian reserve [15, 16]. Altered expression of the two have been found in patients with premature ovarian insufficiency (POI), DOR and infertility [14–16, 21]. Moreover, for patients undergoing IVF-ET treatment, *GDF9* and *BMP15* in follicular fluid (FF) and GCs have been associated with poorer oocyte quality and outcome of treatment [22], hence were proposed as biomarkers for the potential of oocyte development [22, 23].

Considering the important roles of *GDF9* and *BMP15* in folliculogenesis, this prospective study was designed to analyze the expression of *GDF9* and *BMP15* in the FF and GCs from patients with POR from more homogeneous age groups. Identification of the underlying mechanism may facilitate design of individualized treatment protocols for subgroups of patients with POR.

## Methods

### Population

This study was registered with the Chinese Clinical Trial Registry Center (Registration No. ChiCTR1800016107) and approved by the Medical Ethics Committee of Sichuan Provincial Women’s and Children’s Hospital. All patients had given written informed consent. The study also conformed to the Declaration of Helsinki for Medical Research involving Human Subjects (2013 revision).

Ethnic Han Chinese patients diagnosed with POR by the Bologna criteria [1] were enrolled from May 2018 to October 2019 and divided into three groups: group A (< 35 year old), group B (35–40 year old), and group C (> 40 year old). All patients underwent IVF-ET treatment. Patients were excluded from the study should they meet any of the following criteria: (1) Congenital uterine malformation, endometriosis, polycystic ovarian syndrome, intrauterine adhesion, single ovary; (2) Systemic lupus erythematosus and/or sicca syndrome; (3) Uncontrolled endocrinopathy such as diabetes, hyper/hypothyroidism, and hyperprolactinemia; (4) Abnormal chromosomal karyotype; (5) Controlled ovarian stimulation (COS) in the past 3 months; and (6) Intracytoplasmic sperm injection (ICSI) cycle due to male factor infertility. Medical history was taken for all participants, including regularity of menstrual cycle, duration of infertility and pre-treatment protocols. Height and body weight (with shoes and heavy clothing taken off) were measured. Body weight index (BMI) was calculated as weight divided by height squared (kg/m^2^).

### COS and IVF procedures

A GnRH antagonist protocol was conducted for all patients. From day 2 or 3 of the cycle, 187.5 ~ 225 IU/d human recombinant follicle stimulating hormone (rFSH) (Gonal-F, Merck Serono, Germany; Puregon®, Merck Sharp & Dohme, USA) and 75 IU/d human menopausal gonadotrophin (hMG) (menotrophin, Livzon Pharmaceutical Group Inc., Zhuhai, China) were injected. The dosage of rFSH and hMG was adjusted according to the ovarian response. Ganirelix (Merck Sharp & Dohme, USA) was administered should one of the following criteria be met: serum estradiol (E_2_) > 300 pg/ml, follicle diameter > 14 mm, luteinizing hormone (LH) > 10 IU/L. When the leading follicle reached 18 mm in diameter, 10,000 IU of urinary human chorionic gonadotrophin (uhCG) (Livzon Pharmaceutical Group Inc., Zhuhai, China) was injected to trigger the ovulation. Oocytes were retrieved by trans-vaginal ultrasound guidance within approximately 36 h after the trigger, and follicle flushing was not used.

Oocytes were fertilized by conventional IVF for 4 ~ 6 h. Mature oocyte was defined as being at the metaphase II (MII) stage with the first polar body visible in the cytoplasm. 17 ~ 18 h after the IVF, normal fertilized oocyte was confirmed should it contain two pronuclei (2PN). Cultured embryos were evaluated on day 3 based on the number of blastomeres and degree of fragmentation. Embryos of grade A ~ C on day 3 were defined as transplantable embryos [24]. One or two transplantable embryos were transferred. Luteal phase support was started on the oocyte retrieval day with the injection of 60 mg/d progesterone oil (Zhejiang Xianju Pharmaceutical Co., Ltd. Taizhou, China) or vaginal progesterone (Crinone 8% gel, Merck, Germany). The reasons for canceled cycles included follicular growth failure (10 days after COS, leading follicle diameter < 10 mm), failed oocyte retrieval at the time of follicle aspiration, no transplantable embryos (no mature oocyte, abnormal fertilization or cleavage), and accumulation of embryos and progesterone > 2.5 ng/ml on the trigger day. Clinical pregnancy was defined as detection of embryonic heartbeat. The rates of implantation, clinical pregnancy, multiple pregnancy, and miscarriage were calculated [8].

### Measurement of basal endocrine parameters in serum

Endocrine parameters including E_2_, progesterone (P), total testosterone (TT), prolactin (PRL), FSH and LH were measured with an electrochemiluminescence immunoassay platform (Roche Diagnostics GmbH, Mannheim, Germany). Anti-Müllerian hormone (AMH) was measured with an enzyme-linked immunosorbent assay kit (Guangzhou Kangrun Biotech, Co., Ltd., Guangdong, China). Intra- and inter-assay coefficients for the above variables were set as < 5 and 10%, respectively.

### Collection of FF and GCs

FF samples were carefully collected from follicles with a diameter of ≥18 mm, and centrifuged immediately at 700×g for 5 min. The supernatant was stored at − 80 °C. GCs were obtained by follicular aspiration and isolated from blood cells and cellular debris with a lymphocyte separation medium (Beijing Solarbio Science and Technology Corporation, Beijing, China) by centrifugation at 700×g for 10 min. Residual red blood cells were removed with a red blood cell lysis buffer (Solarbio Science and Technology Corporation, Beijing, China). The GCs were stored at − 80 °C until the time of use. For each patient, the FF and GCs were collected from all follicles and pooled as one sample.

### Determination of GDF9 and BMP15 in FF by ELISA

The concentrations of GDF9 and BMP15 in FF were measured with a commercial enzyme-linked immunosorbent assay kit (Elabscience Biotechnology Co., Ltd., Wuhan, China) by following the manufacturer’s instructions. The standard product in GDF9 assay kit was human GDF9 recombinant protein expressed in *E. coli*, and the sequence was Gly320-Arg454. The kit has been crossed experimented with human GDF-2, GDF-7, GDF-10, GDF-11, GDF-15 and BMP-15, with the crossover rates being 0.74, 0.61, 0.78, 0.65, 0.72 and 0.41%, respectively. The standard product of the BMP15 assay kit was human BMP15 recombinant protein expressed in *E. coli*, and the sequence was Gln268-Arg392. The kit has been crossed experimented with human BMP-1, BMP-2, BMP-4, BMP-6, BMP-7 and GDF9, with the crossover rates being 0.53, 0.92, 0.79, 0.56, 0.74 and 0.38%, respectively. The standard use of the two kits was to make serial dilutions, i.e., 1000, 500, 250, 125, 62.5, 31.25, 15.63, 0 pg/ml, of a working solution of 1000 pg/ml before the measurement. The FF samples were thawed and mixed together, and 100 μl FF was added to each well in duplicate. The absorbance value was measured at 450 nm with a Perlong DNM-9602G microplate spectrophotometer (Perlong New Technology Co., Ltd., Beijing, China). The sensitivity of the assay was set as 9.38 pg/ml. The detection range of GDF9 and BMP15 was set as 15.36 ~ 1000 pg/ml. The concentration of GDF in some FF samples were above the range. Such samples were diluted 2 times, and 100 μl diluted FF was then added to each well. The final concentration was calculated by multiplying the detection value with the dilution factor.

### Determination of mRNA expression in GCs by quantitative real-time polymerase chain reaction (qRT-PCR)

Total RNA was isolated from GCs with a RNAprep Pure Micro Kit (Tiangen Biotech Co., Ltd., Beijing, China). The purity and concentration of RNA were determined with Nanodrop-2000 (Thermo Fisher Scientific, Waltham, Massachusetts, USA) under the absorbance of 260 nm/280 nm. The RNA was reversely transcribed into cDNA using a PrimeScript™ RT Reagent Kit with gDNA Eraser (TaKaRa, Tokyo, Japan). The cDNA was then amplified using TB Green™ Premix Ex Taq™ II (TaKaRa, Tokyo, Japan) by qRT-PCR in triplicate. The PCR conditions were 95 °C for 30 s, followed by 40 cycles of 95 °C for 10 s, 60 °C for 30 s, and 65 °C for 5 s. Formation of a single product was verified with a melting curve method. *GAPDH*, *β-actin* and *HPRT* were used as internal controls. The mRNA levels of the target genes were calculated with the 2^-ΔCT^ method and expressed as fold change relative to the geometric means of the three internal controls. All PCR reactions were conducted in triplicate. Primers used in the qRT-PCR are shown in Table 1.

**Table 1:** Sequences of primers used in qRT-PCR

Gene	Primer (5′ → 3′)	Annealing temperature (°C)	Efficiency
*GDF9*	F: TGGAGCATCCTTCAGCAC R: GCAGCCTCTTCTCCCACA	57.2	98.8%
*BMP15*	F: TTTACCGCCATCATCTCCAA R: TTTCCAAGCGTTAGACATCA	53.4	91.9%
*GAPDH*	F: ACGGATTTGGTCGTATTGGG R: CGCTCCTGGAAGATGGTGAT	57.4	101.6%
*β-actin*	F: GACAGGATGCAGAAGGAGAT R: CTGCTTGCTGATCCACATCT	53.5	99.6%
*HPRT*	F: CCATTCCTATGACTGTAGAT R: CCAGTTAAAGTTGAGAGATCAT	51.8	96.5%

### Determination of GDF9 and BMP15 in GCs by Western blotting

GCs were lysed in RIPA lysis buffer (KeyGen Biotech Co., Ltd., Nanjing, China) containing the Halt™ Protease Inhibitor Cocktail (Invitrogen, Karlsruhe, Germany). ABCA protein quantitative kit (Pierce, Thermo Fisher Scientific, Rockford, IL, USA) was used for determining the protein concentration. The total proteins (60 μg/lane) were subjected to 10% SDS-PAGE and transferred onto a polyvinylidene fluoride (PVDF) membrane (EMD Millipore, Billerica, MA, USA). The membrane was then blocked in TBST (Tris-buffered saline with Tween-20) containing 5% BSA (Bio-Rad, Hercules, CA, USA) at room temperature for 1 h, and incubated with primary antibody overnight at 4 °C. The primary antibodies used in this study have included GDF9 (1:500, Beyotime Biotechnology Co., Ltd., Shanghai, China), BMP15 (1:500, Beyotime Biotechnology Co., Ltd.), and GAPDH (1:2000, Bioss, Beijing, China). After washed with TBST for three times, the membranes were incubated with secondary antibodies for 2 h at room temperature. The protein was then detected with a SuperSignal® West Pico Trial Kit (Thermo Scientific Pierce, IL, USA). Band intensity was measured with a Gel Doc XR densitometer (Bio-Rad, Hercules, CA, USA) and normalized with that of the internal control.

### Statistical analysis

Data were analyzed with SPSS 17.0 software (SPSS Inc., Chicago IL, USA). Continuous variables were expressed as mean ± standard deviation (SD). The Kolmogorov-Smirnov test was used to assess the normality of data distribution. Continuous variables with normal distribution were compared using one-way ANOVA with post hoc Bonferroni test. Categorical data were compared using Chi-squared test. Pearson’s correlation was used to assess the correlation between the relative mRNA levels in GCs and protein concentrations in FF. Significance level was set as < 0.05, and two-tailed test was used for all hypothesis tests. Linear regression equation was established for the expression levels of GDF9 against BMP15 mRNA in each sample.

## Results

### Baseline characteristics of the patients

Baseline characteristics of the patient are shown in Table 2. The average ages of the three groups have differed significantly (*P* <  0.05). Compared with group A, the duration of infertility was significantly longer in group C (*P* <  0.05). The abnormal menstrual cycle rate, BMI, AFC, AMH and basal hormone levels did not significantly differ among the three groups (*P* > 0.05).

**Table 2:** Baseline characteristics of the patients

	group A (***n*** = 63)	Group B (***n*** = 65)	Group C (***n*** = 68)
Age (years) ^abc^	30.65 ± 2.57	37.37 ± 1.53	42.59 ± 1.70
Abnormal menstrual cycle	34.92% (22/63)	36.92% (24/65)	35.29% (24/68)
Duration of infertility (years) ^b^	3.32 ± 2.21	4.03 ± 3.05	4.68 ± 3.00
BMI (kg/m^2^)	22.08 ± 2.62	22.21 ± 2.00	22.44 ± 2.62
AFC	4.05 ± 1.43	3.91 ± 1.69	3.94 ± 1.68
FSH/LH ratio	2.68 ± 1.29	2.76 ± 1.71	2.99 ± 1.21
E_2_ (pg/ml)	76.12 ± 34.71	77.49 ± 52.67	85.08 ± 50.88
P (ng/ml)	0.77 ± 0.35	0.78 ± 0.33	0.84 ± 0.52
TT (ng/ml)	0.37 ± 0.15	0.36 ± 0.15	0.39 ± 0.10
AMH (ng/ml)	0.80 ± 0.39	0.78 ± 0.24	0.73 ± 0.39
PRL (ng/ml)	199.84 ± 39.35	205.58 ± 58.49	197.26 ± 59.45

Data are presented as mean ± SD or percentage (number). ***Abbreviations:*** *BMI* Body mass index, *AFC* Antral follicle count, *FSH* Follicle stimulating hormone, *LH* Luteinizing hormone, *E*_*2*_ Estradiol, *P* Progesterone, *TT* Total testosterone, *AMH* Anti-Müllerian hormone, *PRL* Prolactin. The rate of abnormal menstrual cycle was compared with Chi-square test. *P* <  0.05 was considered as statistically significant

^a^ *P* < 0.05, group A vs. group B

^b^ *P* < 0.05, group A vs. group C

^c^ *P* < 0.05, group B vs. group C

### Decline of oocyte quality and IVF outcome with increased age

In total thirty-one cycles were canceled. The main reasons included follicular growth failure (10 cycles), failed oocyte retrieval at the time of follicle aspiration (6 cycles), no transplantable embryos (5 cycles), accumulation of embryos (5 cycles), and progesterone > 2.5 ng/ml on the trigger day (5 cycles). Compared with group C, groups A and B had significantly more 2PN oocytes, transplantable embryos, and higher rates of implantation and clinical pregnancy (*P* < 0.05). The dosage of rFSH, duration of COS, endometrial thickness and E_2_ levels on the trigger day, the number of retrieved oocytes, MII oocytes and embryos per ET, and the rates of miscarriage and multiple pregnancy did not significantly differ among the three groups (*P* > 0.05) (Table 3).

**Table 3:** COS, IVF outcomes and biochemical markers in FF

	group A (***n*** = 63)	Group B (***n*** = 65)	Group C (***n*** = 68)
COS			
Dosage of rFSH (IU)	2476.31 ± 379.34	2556.69 ± 419.57	2586.90 ± 587.63
Duration of COS (d)	9.89 ± 2.85	9.68 ± 2.22	9.33 ± 2.34
E_2_ on trigger day (pg/mL)	756.95 ± 191.65	740.23 ± 180.47	721.03 ± 151.62
Endometrial thickness (mm)	9.10 ± 1.89	8.95 ± 1.91	8.81 ± 1.61
Number of oocytes retrieved	2.98 ± 0.67	2.82 ± 0.87	2.68 ± 0.90
Number of MII	2.54 ± 0.94	2.43 ± 0.79	2.37 ± 0.74
Number of 2PN ^ab^	2.08 ± 0.93	1.87 ± 0.87	1.36 ± 0.58
Number of transplantable embryos ^ab^	2.02 ± 0.57	1.85 ± 0.71	1.22 ± 0.49
Cancel cycle rate (%)	11.11% (7/63)	12.31% (8/65)	23.53% (16/68)
ET			
Number of embryos per ET	1.43 ± 0.50	1.44 ± 0.50	1.33 ± 0.47
Implantation rate (%) ^ab^	30.00% (24/80)	24.39% (20/82)	10.14% (7/69)
Clinical pregnancy rate (%) ^ab^	37.50% (21/56)	29.82% (17/57)	11.54% (6/52)
Miscarriage rate (%)	9.52% (2/21)	23.53% (4/17)	33.33% (2/6)
Multiple pregnancy rate (%)	14.29% (3/21)	17.65% (3/17)	16.67% (1/6)
In FF			
*GDF9* (pg/ml) ^abc^	1020 ± 117	904 **±** 70	671 ± 53
*BMP15* (pg/ml) ^abc^	666 ± 40	643 **±** 46	420 ± 40

Data are presented as mean ± SD or percentage (number). ***Abbreviations:*** *COS* Controlled ovarian stimulation, *E*_*2*_ Estradiol, *ET* Embryos transferred, *FF* Follicle fluid, *GDF9* Growth differentiation factor 9, *BMP15* Bone morphogenetic protein-15. Chi-squared test was used to compare the rates of maturation, fertilization, cleaved embryo, higher quality embryo, cancel cycle, implantation, clinical pregnancy, miscarriage and multiple pregnancy between the two groups. *P* < 0.05 was considered as statistically significant

^a^ *P* < 0.05, group A vs. group C

^b^ *P* < 0.05, group B vs. group C

^c^ *P* < 0.05, group A vs. group B

### Decreased expression of GDF9 and BMP15 in FF with increased age

As shown in Table 3, the concentration of GDF9 (1012 ± 117 pg/ml vs. 904 ± 70 pg/ml vs. 671 ± 53 pg/ml) and BMP15 (666 ± 40 pg/ml vs. 643 ± 46 pg/ml vs. 420 ± 40 pg/ml) in FF have differed significantly between the three groups (*P* < 0.05), with an obvious trend of decline with increased age.

### Decreased expression of GDF9 and BMP15 in GCs with increased age

**Fig. 1:**
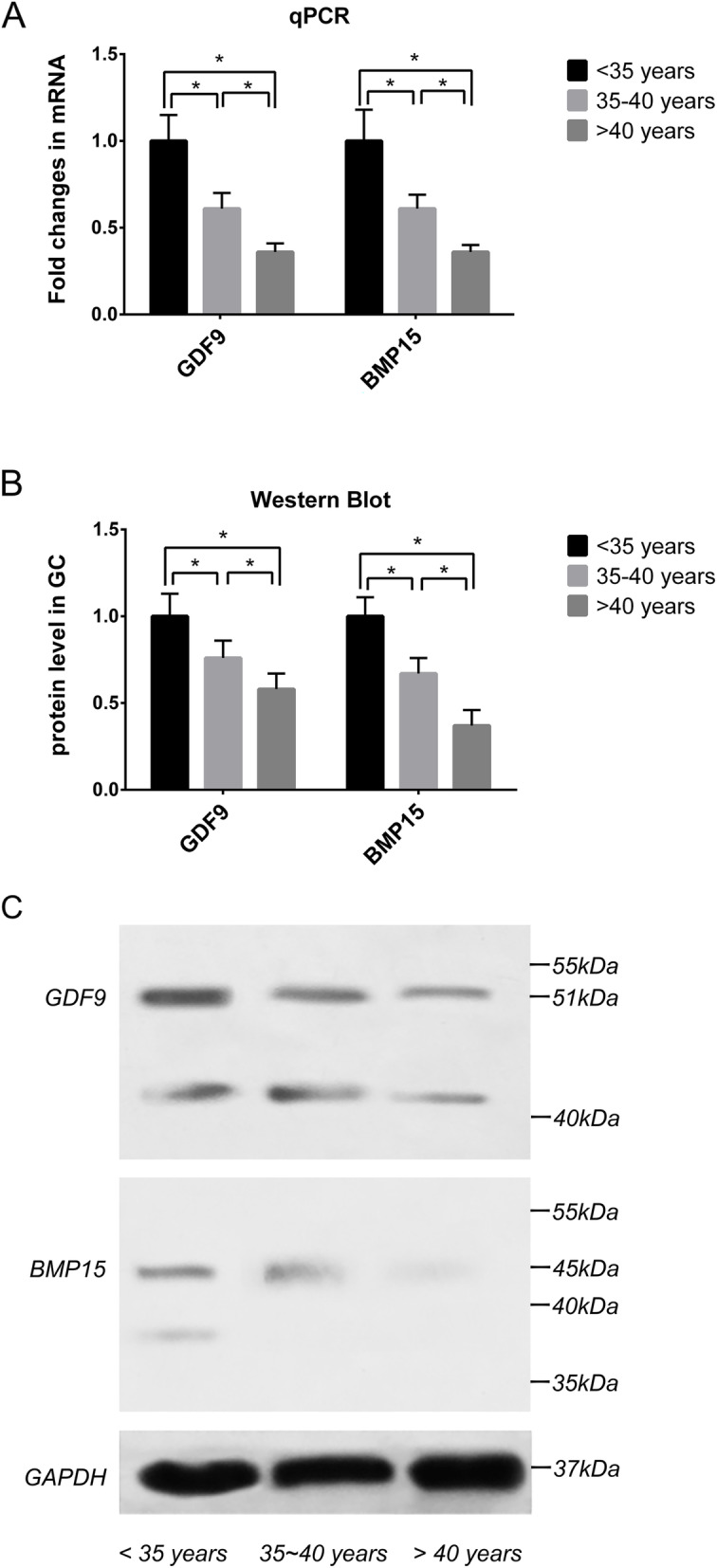
The gene and protein levels of *GDF9* and *BMP15* in GCs were decreased with increasing age. **a** The relatively mRNA levels of *GDF9* and *BMP15* were significantly different between the three groups (*P* < 0.05). They were decreased with increasing age. *GAPDH*, *β-actin* and *HPRT* were used as the internal controls. **b** The relatively protein levels of *GDF9* and *BMP15* were significantly different between the three groups (*P* < 0.05). They were decreased with increasing age. GAPDH was used as internal control. **c** A single band at 51 kDa represented *GDF9*, and 45 kDa represented *BMP15* by western blot, respectively

Only GCs in FF with oocyte were detected for mRNA and protein. Therefore, there were 61, 60 and 59 samples from group A, group B and group C, respectively. The CT values of housekeeping genes did not differ significantly among the three groups (*P* > 0.05). As shown in Fig. 1a and b, the expression of *GDF9* and *BMP15* in GCs, at the levels of both mRNA and protein, have differed significantly between the three groups (*P* < 0.05). Both showed a trend of decline with increased age. As shown in Fig. 1c, the single band at 51 kDa represented *GDF9*, while the single band at 45 kDa represented *BMP15*.

As shown in Table 4, the relative protein levels in GCs was positively correlated with the relative mRNA levels in GCs and protein concentrations in FF by Pearson’s correlation analysis (*P* < 0.05).

**Table 4:** Correlation between the proteins and mRNA levels

relative protein levels in GCs	relative mRNA levels in GCs		protein concentration in FF	
	*r*	*P*	*r*	*P*
GDF9	0.704	< 0.001	0.501	0.001
BMP15	0.737	< 0.001	0.701	< 0.001

### The ratio of GDF9/BMP15 mRNA levels

The ratio of GDF9/BMP15 mRNA levels of the three groups were compared using one-way ANOVA. The ratio of GDF9/BMP15 mRNA levels for groups A, B and C were 1.23 (1.00–1.35), 1.23 (1.08–1.38), and 1.20 (1.09–1.33), respectively. No significant difference was detected between the groups (*P* > 0.05). Linear regression equation for the mRNA levels of GDF9 against BMP15 in each sample was established as *y* = 1.097*x* + 0.047.

## Discussion

We have proven through this study that the oocyte quality and IVF outcome will decrease with increased age among patients with POR, in particular those over 40, which also correlated with decline of *GDF9* and *BMP15* expression in FF and GCs. The ratio of GDF9/BMP15 mRNA levels has remained to be relatively constant, and the protein and mRNA levels were positively correlated in both FF and GCs.

Although the quantity of oocyte was poor in all groups in this study, the numbers of normal fertilized oocytes and transplantable embryos have decreased steeply to 1.36 and 1.22 in poor responders over 40, respectively. And the oocyte quality is one of the most critical factors for pregnancy. Similar to previous reports, the rates of implantation and clinical pregnancy in elder patients (> 40) have decreased to 10.14 and 11.54%, respectively [7, 8]. This seems to have reflected the poor quantity and quality of the oocytes from elder poor responders whom may benefit less from IVF treatment. For poor responders between 35 and 40, the rates of implantation and clinical pregnancy were 24.39 and 29.82%, respectively. Both were lower than those of under 35, albeit with no statistical significance. Researchers have previously proposed that 35–37 year of age to be the threshold for decreased rate of euploidy embryos and live births [25]. Such patients should therefore consider IVF treatment earlier. Meanwhile, younger poor responders (< 35) still have reasonable number of transplantable embryos (2.02 ± 0.57) and pregnancy rate (37.50%), and should be actively treated. Of note, the miscarriage rate may also rise with increased age [8]. The underlying mechanisms for age-related decline of oocyte quality and IVF outcome are variable, including chromosomal aberration in oocytes and embryos due to advanced maternal age [26–28]. The autocrine function of oocytes in POR is also important but not yet fully understood.

GDF9 and BMP15 secreted by the oocyte play an important role in the cross-talk between cumulus GCs and the oocytes [22]. The functions of GDF9 and BMP15 in follicular development include suppressing GCs apoptosis and promoting cell proliferation [29], enhancing the effect of FSH and insulin-like growth factor-I (IGF- I) on GCs, then providing more E_2_ and glycolysis for oocyte [30, 31]. GDF9 and BMP15 can also prevent premature luteinization and promote normal expansion of cumulus cells till the LH surge [32, 33]. In turn, GCs are necessary to nourish the oocytes and promote their maturation [17, 34]. Therefore, altered expression and/or function of GDF9 and BMP15 may result in abnormal folliculogenesis and poor oocyte quality.

Declined expression of the *GDF9* and *BMP15* genes has previously been discovered among patients with DOR [21, 35]. In this study, we further proved that such decline is associated with increased age, particularly for those over 40. FF and GCs form the microenvironment of the oocytes, and certain components of them may reflect the metabolism and endocrine status of the oocyte [34, 36]. In this study, we found that the relative protein levels in GCs have correlated with the relative mRNA levels in GCs and protein concentration in FF. This has reflected the similar changes of the two proteins in FF and GCs, both of which can reflect the status of oocyte. Furthermore, the decline of *GDF9* and *BMP15* expression was accompanied with poor oocyte quality and IVF outcome in elder patients. Compared with group A (< 35), group B (35–40) had significantly lower expression. Their oocyte quality and IVF outcome were also poorer albeit with no statistical significance. Hence, *GDF9* and *BMP15* may provide more sensitive and earlier biomarkers for the fertility of poor ovarian responders. Declined expression of the two may, at least in part, account for the age-related poor oocyte quality in poor responders. Physiologically, *GDF9* and *BMP15* can promote the expansion of cumulus GCs till the LH surge [32, 33]. For elder poor responders, the steep decline in *GDF9* and *BMP15* expression may affect the response of GCs to LH surge. For such patients, exogenous LH in conjunct with rFSH may compensate the insufficient function of LH and improve the outcome of IVF-ET treatment [37].

Of note, the ratio of GDF9/BMP15 mRNA levels in GCs has remained relatively constant among the three groups, and therefore may not be associated with the onset of early DOR. Crawford et al. [38] also found the ratio of GDF9/BMP15 mRNA to be strongly reserved in each species but differed markedly between species. Oocyte derived from poly-ovulatory species exhibited higher GDF9/BMP15 ratios, e.g., mouse (5.19) and rat (3.65), compared with species with low ovulation rates, e.g., sheep (1.26), cow (0.24) and deer (0.10) [38]. In the present study, the ratio of human GCs (1.20–1.23) was similar to that of low ovulatory species such as sheep (1.26). GDF9 and BMP15 work synergistically to influence the proliferation of GCs, and GCs of each species might have evolved to respond to unique GDF9/BMP15 ratios [39]. Nevertheless, Crawford et al. found the ratio in pig oocytes (poly-ovulatory species) to be also low (0.51) [38]. Therefore, the ratio of GDF9/BMP15 may not be the only factor which can influence the ovulation rate. The underlying mechanism of the GDF9/BMP15 ratio and ovulation rate demands further research.

The limitation of this study lies in its relatively small sample size and lack of livebirth rate due to the limited study period. Moreover, as the main purpose of this study was to evaluate *GDF9* and *BMP15* expression in female age groups, we have excluded ICSI cycles due to male factors. The latter, in particular advanced paternal age, may also affect the embryo quality and outcome of IVF-ET treatment.

## Conclusions

In summary, we have discovered an age-related decline in *GDF9* and *BMP15* expression among patients with POR, which may in part account for the age-related poor oocyte quality and IVF outcome. Identification of the heterogeneity may facilitate design of individualized protocols to attain better treatment outcome and avoid repeated cycles. Larger cohort studies are required to validate the results of this study. Researchers have recently suggested that exogenous recombinant *GDF9* and/or *BMP15* can enhance blastocyst formation during IVF treatment [40, 41]. Should such strategy be adapted for poor ovarian responders, dosages dependent on age and basal levels of *GDF9* and *BMP15* expression may be considered.

## Data Availability

The datasets used and/or analyzed during the current study are available from the corresponding author on reasonable request.

## Abbreviations

*GDF9*: Growth differentiation factor 9
*BMP15*: Bone morphogenetic protein 15
POR: Poor ovarian response
FF: Follicle fluid
GCs: Granulosa cells
IVF-ET: In vitro fertilization and embryo transfer
ELISA: Enzyme-linked immunosorbent assay
qRT-PCR: Quantitative real-time polymerase chain reaction
AFC: Antral follicle count
CLBR: Cumulative live birth rate
DOR: Decreased ovarian reserve
OSFs: Oocyte-secreted factors
TGFβ: Transforming growth factor β
POI: Premature ovarian insufficiency
COS: Controlled ovarian stimulation
ICSI: Intracytoplasmic sperm injection
FSH: Follicle stimulating hormone
hMG: human menopausal gonadotrophin
uhCG: urinary human chorionic gonadotrophin
2PN: Two pronuclei
E_2_: Estradiol
P: Progesterone
TT: Total testosterone
PRL: Prolactin
